# Resource competition modeling suggests hydrogen peroxide determines competitive outcomes among oligotrophic cyanobacteria

**DOI:** 10.1093/ismeco/ycag188

**Published:** 2026-07-01

**Authors:** Donna Katie McCullough, David Talmy

**Affiliations:** Department of Microbiology, University of Tennessee, Knoxville, TN 37996, United States; Department of Microbiology, University of Tennessee, Knoxville, TN 37996, United States

**Keywords:** hydrogen peroxide, resource competition, mathematical modeling, *Prochlorococcus*, ecology, ocean, reactive oxygen species

## Abstract

*Prochlorococcus* is the most numerically abundant photosynthetic organism in the oligotrophic ocean; yet, it is vulnerable to damage by the reactive oxygen species (ROS) hydrogen peroxide (H_2_O_2_). Detoxifying microbes and abiotic decay are thought to mitigate the most harmful impacts of H_2_O_2_, but the ecological impacts of ROS on ocean microbial community composition are not fully understood. Here, we introduce ROS dynamics within a resource competition model to investigate H_2_O_2_ impacts on a community with one catalase negative *Prochlorococcus* analogue, one catalase positive *Synechococcus* analogue, and a heterotrophic bacterium. Model parameters defining resource utilization, H_2_O_2_ detoxification, and H_2_O_2_-mediated cell death are constrained with data from laboratory experiments. With these ecologically realistic parameter values, we investigate the community composition for a range of ammonium and H_2_O_2_ supply rates. In the absence of a heterotrophic bacterium, *Synechococcus*’ modest ability to detoxify H_2_O_2_ facilitates the survival of *Prochlorococcus* under conditions that would otherwise be fatal. However, in the absence of a strong bacterial detoxifier, unrealistically high *Synechococcus* concentrations (>10^6^ mL^−1^) are required for it to coexist with *Prochlorococcus* on a single limiting nutrient. Coexistence among *Prochlorococcus* and *Synechococcus* at ecologically realistic cell densities and H_2_O_2_ concentrations is observed when heterotrophic bacteria with high detoxification rates reach cell densities on the order of 10^5^ cells mL^−1^. Our analysis suggests that environmentally relevant ROS concentrations have the potential to determine whether *Prochlorococcus* and *Synechococcus* coexist on ammonium and points to the importance of ROS supply and degradation for understanding marine cyanobacteria ecology.

## Introduction

**Figure 1:**
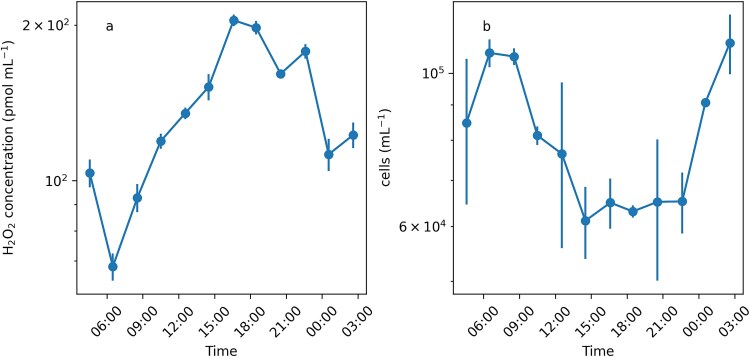
Increases in hydrogen peroxide concentration (a) are associated with decreases in *Prochlorococcus* cell density (b) in the South Pacific. Data represent average values within the mixed layer observed by Morris et al. (2016) [29].

*Prochlorococcus* numerically dominates oligotrophic surface waters [1, 2] and grow alongside their closest extant relative *Synechococcus* [3]. Efficiency of nutrient usage due to genomic and cell streamlining allows *Prochlorococcus* to be the superior competitor for ammonium [4–6], while its limited ability to utilize nitrate [7, 8] allows coexistence with *Synechococcus* [9]. Resource competition theory [10] has formed a basis on which to understand the biogeography of diverse phytoplankton in the global ocean [11]. It is premised on the notion that in steady-state (i.e. when growth balances loss), the dominant organisms are those able to maintain growth at the lowest concentration of limiting nutrient—i.e. their “R*.” Specialization for distinct nutrients is sufficient for competing organisms to coexist [10, 12], but resource competition theory has been expanded to account for alternative explanations for coexistence. For example, inhibition of one species via an external inhibiter molecule has been found to promote stable coexistence on a single nutrient when a competing organism is not susceptible [13–15]. The reactive oxygen species (ROS) hydrogen peroxide is an exogenous toxin with the potential to facilitate this type of coexistence among *Prochlorococcus* and *Synechococcus. Prochlorococcus* lacks the enzyme catalase-peroxidase [16, 17] and as little as 300–400 pmol mL^−1^ H_2_O_2_ can be deadly in monoculture [18, 19], roughly half the H_2_O_2_ that can be produced in *Prochlorococcus*’s environment [19] through a combination of photochemical H_2_O_2_ production from dissolved organic carbon [20, 21], cells [22–24], and periodic rain events [25, 26]. That said, a combination of photochemical degradation [25] and cell-mediated decomposition [27, 28] keep H_2_O_2_ concentration below ~200 pmol mL^−1^, preventing direct *Prochlorococcus* mortality [19, 29]. Therefore, hydrogen peroxide has not been invoked as a primary determinant of cyanobacteria ecology in the oligotrophic gyres. Nevertheless, even within ranges considered permissible, increases in hydrogen peroxide concentration have been observed to co-occur with decreases in *Prochlorococcus* abundance (Fig. 1).

Declines in *Prochlorococcus* concentration in Fig. 1b could be mediated by a variety of factors including grazing and viral lysis. One reason to discount H_2_O_2_ damage as an explanation is that laboratory monocultures indicate *Prochlorococcus* abundance only begins to decline when H_2_O_2_ reaches 300–400 pmol mL^−1^ [18, 19], and the most drastic decline in *Prochlorococcus* abundance in Fig. 1b happens before H_2_O_2_ reaches ~200 pmol mL^−1^ (Fig. 1). However, *Prochlorococcus* populations consist of coexisting ecotypes with differential H_2_O_2_ susceptibility [29, 30], and vulnerable types may be removed by H_2_O_2_ damage. Resource competition models suggest that even small modifications to growth rate can have a profound impact on competitive outcomes [31]. The data in Fig. 1 are not evidence that hydrogen peroxide causes *Prochlorococcus* mortality in the environment. Instead, they motivate the hypothesis that *Prochlorococcus* competitive ability could be negatively impacted by H_2_O_2_ through modest changes in growth rate. Here, we take a resource competition modeling approach to explore the potential for hydrogen peroxide to influence competition and coexistence among *Prochlorococcus* and *Synechococcus*. Our modeled *Prochlorococcus* represents strains with high susceptibility to H_2_O_2_, and *Synechococcus* and bacterial analogues represent H_2_O_2_ resistant populations. Model parameters that define susceptibility to H_2_O_2_ and the ability to detoxify H_2_O_2_ are constrained with data from laboratory assays. Utilizing hydrogen peroxide sensitivities at the edge of the thermal range for two species of high-light-adapted *Prochlorococcus*, we consider competitive outcomes in a range of nutrient and hydrogen peroxide regimes. In doing so, we identify environmentally and ecologically realistic scenarios in which hydrogen peroxide has the potential to determine cyanobacteria community composition. Our analysis has implications for models of *Prochlorococcus* and *Synechococcus* ecology that to the best of our knowledge do not assess impacts of ROS on biogeochemical functions.

## Materials and methods

Here, we provide a description of the ecological model, our approach to model parameterization, and obtaining model solutions.

### Model description

The dynamics of a dissolved ammonium (*N*, mM), catalase negative *Prochlorococcus* (*P*, cells mL^−1^), catalase positive *Synechococcus* (*S*, cells mL^−1^), and H_2_O_2_ (*H*, pmol mL^−1^) were modeled with a set of coupled ordinary differential equations:


(1.1) \begin{eqnarray*} \frac{dN}{dt}=\underset{\begin{array}{c} ammonium\\{} supply\end{array}}{\underbrace{\lambda_n}}\!\!-\kern0.6em \underset{\begin{array}{c} nutrient\\{} uptake\end{array}}{\underbrace{Q_{n,p}\frac{\ {\mu}_{m,p}N}{N+{K}_{s,p}}P}}\kern0.75em -\kern0.75em \underset{\begin{array}{c}\ nutrient\ \\{} uptake\end{array}}{Q_{n,s}\underbrace{\frac{\ {\mu}_{m,s}N}{N+{K}_{s,s}}S}}\,\,\,-\!\!\!\underset{\begin{array}{c}\ abiotic\\{} ammonium\\{} loss\end{array}}{\underbrace{\delta_nN}} \end{eqnarray*}

(1.2) \begin{eqnarray*} \frac{dP}{dt}\kern0.5em =\kern0.5em \underset{\begin{array}{c} nutrient\\{} uptake\end{array}}{\underbrace{\frac{\ {\mu}_{m,p}N}{N+{K}_{s,p}}P}}\kern1.5em -\underset{\begin{array}{c}H2O2\ mediated\\{} mortality\\{}\ \end{array}}{\underbrace{k_{dam} PH}}-\underset{\begin{array}{c}\ background\\{} mortality\end{array}}{\underbrace{\delta_pP}} \end{eqnarray*}

(1.3) \begin{eqnarray*} \frac{dS}{dt} = \kern1em \underset{\begin{array}{c}\ nutrient\ \\{} uptake\end{array}}{\underbrace{\frac{\ {\mu}_{m,s}N}{N+{K}_{s,s}}S}}\kern0.75em -\kern0.5em \underset{\begin{array}{c}\ background\\{} mortality\end{array}\ }{\underbrace{\delta_sS}} \end{eqnarray*}

(1.4) \begin{eqnarray*} \frac{dH}{dt\ }\kern1.5em =\underset{\begin{array}{c}H2O2\ \\{} supply\end{array}}{\underbrace{\lambda_h}}\kern1.75em -\underset{\begin{array}{c} syn\\{} detox-\\{} ification\end{array}}{\underbrace{\phi_s SH\kern0.5em }}\kern1.5em -\underset{\begin{array}{c} het\\{} detox-\\{} ification\end{array}}{\underbrace{\phi_b{B}^{\ast }H\kern0.75em }}-\underset{\begin{array}{c}\kern0.5em abiotic\ \\{} decay\ \end{array}}{\underbrace{\ {\delta}_hH\ }} \end{eqnarray*}

All parameter definitions are provided in Table 1. In Equations 1.1–1.4, ammonium is supplied at rate ${\lambda}_n$ (mM day^−1^) and has an abiotic loss rate ${\delta}_n$ (day^−1^). Cyanobacteria are obligately autotrophic, and their ability to take up organic substrates is neglected. Ammonium uptake is assumed to follow Michaelis–Menten [32, 33] kinetics with maximal uptake rate ${\mu}_{m,x}$ (day^−1^) and half-saturation constant ${K}_{s,x}$ (mM) with *x* denoting traits for either *Prochlorococcus* or *Synechococcus*. Hydrogen peroxide is assumed to cause *Prochlorococcus* cell death according to mass action coefficient ${k}_{dam}$, and cell mortality that is independent of hydrogen peroxide is represented as ${\delta}_x$ (day^−1^). Hydrogen peroxide is supplied at rate ${\lambda}_h$ (pmol mL^−1^ day^−1^), decays abiotically at rate ${\delta}_h$ (day^−1^), and is detoxified by cells according to mass action coefficient ${\phi}_s$ for *Synechococcus* and ${\phi}_b$ for heterotrophic bacteria (both mL cell^−1^ day^−1^). The heterotrophic bacteria cell density ${B}^{\ast }$ (mL^−1^) is assumed to be constant, which neglects the potential for bacteria to compete for ammonium [34]. Expansion to account for the diverse substrates that bacteria feed upon would drastically expand the complexity of the model and would require quantitative constraint on bacterial resource affinities that to the best of our knowledge are not readily available.

**Table 1:** Competition model parameter and variable definitions and units.

Symbol	Description	Units	Notes
$N$	Ammonium concentration	mM	State variable
$P$	Cell abundance of *Prochlorococcus*	cells mL^−1^	State variable
$S$	Cell abundance of *Synechococcus*	cells mL^−1^	State variable
$H$	Hydrogen peroxide concentration	pmol mL^−1^	State variable
${\mu}_{m,x}$	Maximal growth rate of plankton, *x = P, S*	day^−1^	[0.63, 0.75] [61, 62]
${K}_{s,x}$	Half-saturation constant of plankton, *x = P, S*	mM	[5.19 × 10^−6^, 2.4 × 10^−5^] from allometric scaling: 0.17 V_x_^0.27^, where V_x_ is spherical volume of cells with radius 0.5 and 0.3 for S and P, respectively [63]
${Q}_{n,x}$	Nitrogen quota, *x = P, S*	mmol cell^−1^	[6.7 × 10^−10^, 1.4 × 10^−9^] for *Prochlorococcus* MED4 and *Synechococcus* WH8102 [64]
${\delta}_x$	Background mortality rate of plankton*, x = S, P*	day^−1^	[0.1, 0.1] assumed
${k}_{dam}$	Hydrogen peroxide cell-specific mortality rate	mL pmol^−1^ day^−1^	0.002, see Supplemental Information Section S1
${\lambda}_n$	Nutrient supply rate	mM day^−1^	Varied within [1 × 10^−4^, 5 × 10^−4^]
${\delta}_n$	Abiotic nutrient loss rate	day^−1^	0.1 assumed
${\lambda}_h$	Hydrogen peroxide supply rate	pmol mL^−1^ day^−1^	Varied within [0, 500]
${\delta}_h$	Abiotic hydrogen peroxide decay rate	day^−1^	0.001 [37]
${\phi}_s$	*Synechococcus* cell-specific rate of H_2_O_2_ detoxification	mL cell^−1^ day^−1^	1.76 × 10^−6^ [37]
${\phi}_b$	Heterotrophic bacteria cell-specific rate of H_2_O_2_ detoxification	mL cell^−1^ day^−1^	1.3 × 10^−5^ [37]
${B}^{\ast }$	Heterotrophic bacteria cell concentration	cells mL^−1^	Varied within [1 × 10^4^, 5 × 10^5^]

The model in Equations 1.1–1.4 makes several simplifying assumptions. For example, *Synechococcus* and *Prochlorococcus* are thought of as bulk representatives of diverse ecotypes and their potential for mixotrophic metabolism [35] is neglected. Losses associated with grazing and viral lysis are encapsulated within the “background” mortality terms for *P* and *S*, and detoxification by picoeukaryotes [18] is neglected. A more complex analysis assessing the potential for these assumptions to modify our results is beyond the scope of the present study, but we describe future studies that could address these limitations in the discussion.

### Model parameterization and experimental approach

The mathematical properties (e.g. stable-states and system stability) of models structurally like Equations 1.1–1.4 have been analyzed extensively by previous authors [13, 14, 36], but to the best of our knowledge, have not been used to examine marine microbial community structure using ecologically realistic parameter values. Here, we derived equilibrium states and conducted numerical simulations to evaluate if these stable states were reached utilizing the parameter values listed in Table 1.

The major goal of our analysis was to evaluate the impact of hydrogen peroxide supply and detoxification on cyanobacteria community composition and dynamics. To quantify the differential impacts of *Synechococcus*, heterotrophic bacteria, and abiotic degradation on *Prochlorococcus* abundance, we examined three special cases: first, control simulations were performed by neglecting all sources of biotic detoxification. Specifically, detoxification by *Synechococcus* was neglected by setting ${\phi}_s=0$ in Equation 1.4, and heterotrophic bacteria detoxification was neglected by setting ${B}^{\ast }=0$, also in Equation 1.4. Second, to isolate the impact of *Synechococcus* detoxification, ${\phi}_s$ was set to the value previously derived from laboratory experiments [37]. Finally, the impact of heterotrophic bacteria detoxification was explored by varying bacterial cell density ${B}^{\ast }$ within the range reported in Table 1. In all cases, environmental variation in nutrient and hydrogen peroxide supply was simulated by repeating simulations over ranges of ${\lambda}_n$ and ${\lambda}_h$ in Table 1. Sensitivity analyses were performed by repeating competition experiments over these ranges of ${\lambda}_n$ and ${\lambda}_h$, assuming a 50% decrease and increase in the default value listed in Table 1 for four key parameters: (i) the rate of H_2_O_2_-induced cell death (${k}_{dam}$), (ii–iii) *Synechococcus* and heterotrophic bacteria cell-specific detoxification rates (${\phi}_s$ and ${\phi}_b$, respectively), and (iv) the rate of background mortality for *Synechococcus* and *Prochlorococcus* (${\delta}_p$).

Numerical simulations were performed in Python using the NumPy and SciPy packages. All code and output are available in a publicly accessible GitHub repository: https://github.com/dtalmy/h2o2comp (commit hash dac67e9). All simulations assumed the same initial conditions: 1 × 10^5^, 1 × 10^5^, 0.1, 1 for P, S, N, and H, respectively. Extensive sensitivity experiments indicated that whether the system converged to equilibrium values did not depend on these specific choices. In all cases, the system was simulated for the time necessary for it to reach equilibrium states.

## Results

Solving Equations 1.2 and 1.3 in equilibrium, we find that *Prochlorococcus* and *Synechococcus* populations can only be maintained with nutrient concentration


(2) \begin{eqnarray*} {N}^{\ast }=\frac{K_{s,p}\left({\delta}_p+{k}_{dam}{H}^{\ast}\right)}{\ {\mu}_{m,p}-\left({\delta}_p+{k}_{dam}{H}^{\ast}\right)} \end{eqnarray*}

for *Prochlorococcus* and


**Figure 2:**
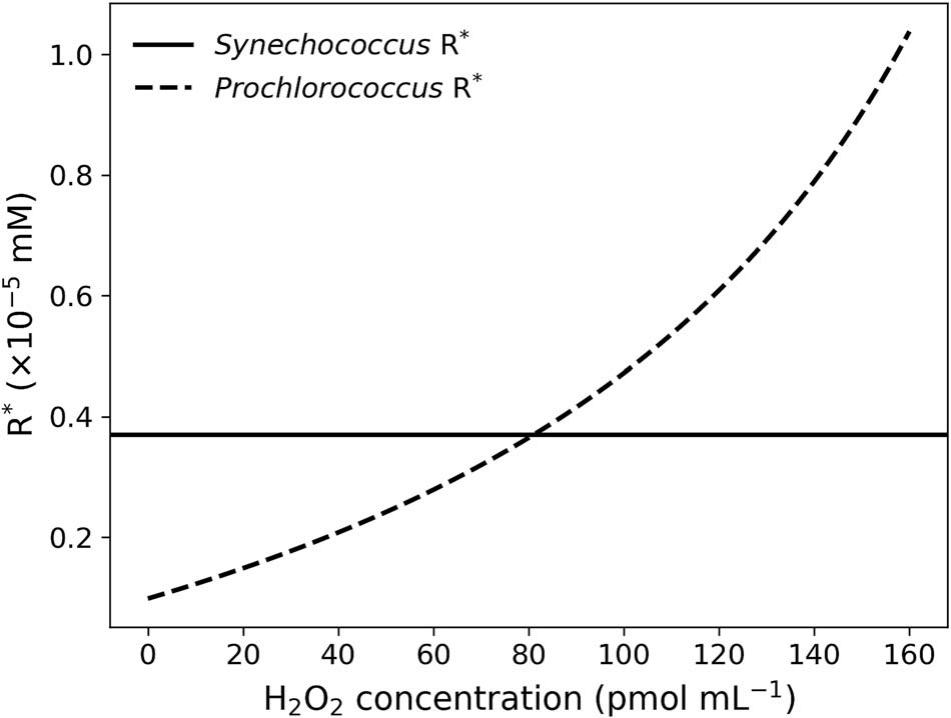
Environmentally relevant hydrogen peroxide concentration increases *Prochlorococcus*’ minimal nutrient requirement. Shown are the nutrient concentrations for *Prochlorococcus* and *Synechococcus* to maintain equilibrium cell densities, found by setting Equations 1.2 (*Prochlorococcus*) and 1.3 (*Synechococcus*) equal to zero and solving for N.

(3) \begin{eqnarray*} {N}^{\ast }=\frac{K_{s,s}{\delta}_s}{\ {\mu}_{m,s}-{\delta}_s} \end{eqnarray*}

for *Synechococcus*. A central tenet of resource competition theory [10] is that the organism with the lower minimal resource requirement will outcompete the other for a shared nutrient. In Fig. 2, we show that, in the absence of hydrogen peroxide, *Prochlorococcus* has the lower minimal resource requirement and is anticipated to be the superior nutrient competitor. However, as the hydrogen peroxide concentration increases, *Prochlorococcus’* minimal resource requirement increases, becoming larger than that of *Synechococcus’*. Therefore, increased hydrogen peroxide concentration has the potential to prevent *Prochlorococcus* from being the superior nutrient competitor. Interestingly, the hydrogen peroxide concentration required to shift competitive outcomes is relatively modest (~80 pmol mL^−1^) and well within ranges reported in marine environments dominated by these cyanobacteria [25, 29].

**Figure 3:**
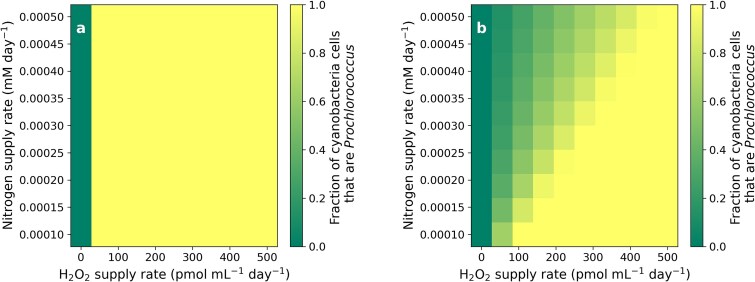
Exogenous detoxification of hydrogen peroxide promotes coexistence among *Prochlorococcus* and *Synechococcus* when grown on a single limiting nutrient. (a) Cyanobacteria taxonomic composition with solutions to equations 1.1–1.4 without any biological detoxification of hydrogen peroxide. Increased hydrogen peroxide supply causes a switch from a system dominated by *Prochlorococcus* (green stripe) to a system dominated by *Synechococcus* (yellow region) (b) same as (a) but *Synechococcus* detoxifies hydrogen peroxide, and there is a region of apparent coexistence at high nutrient concentrations and low hydrogen peroxide supply rate.

To evaluate whether hydrogen peroxide could modify competitive outcomes, we conducted numerical competition experiments, solving Equations 1.1–1.4 across a range of nutrient and hydrogen peroxide supply rates. In Fig. 3, we show results of this competition in a control case with no biological detoxification and a low, baseline value for abiotic decay (Table 1 and Fig. 3a) compared with a case where *Synechococcus* actively detoxify hydrogen peroxide (Fig. 3b). The simulations suggest that in the absence of hydrogen peroxide, *Prochlorococcus* outcompetes *Synechococcus* (green strip in Fig. 3a), and the opposite is true when hydrogen peroxide is supplied, regardless of nutrient enrichment (yellow block, Fig. 3a). The same basic pattern exists when *Synechococcus* degrades hydrogen peroxide, except that there is a region of coexistence at high nutrient and low hydrogen peroxide supply rates (Fig. 3b). Thus, this model reveals the potential for nutrients and hydrogen peroxide to govern *Prochlorococcus* and *Synechococcus* abundances in the ocean.

**Figure 4:**
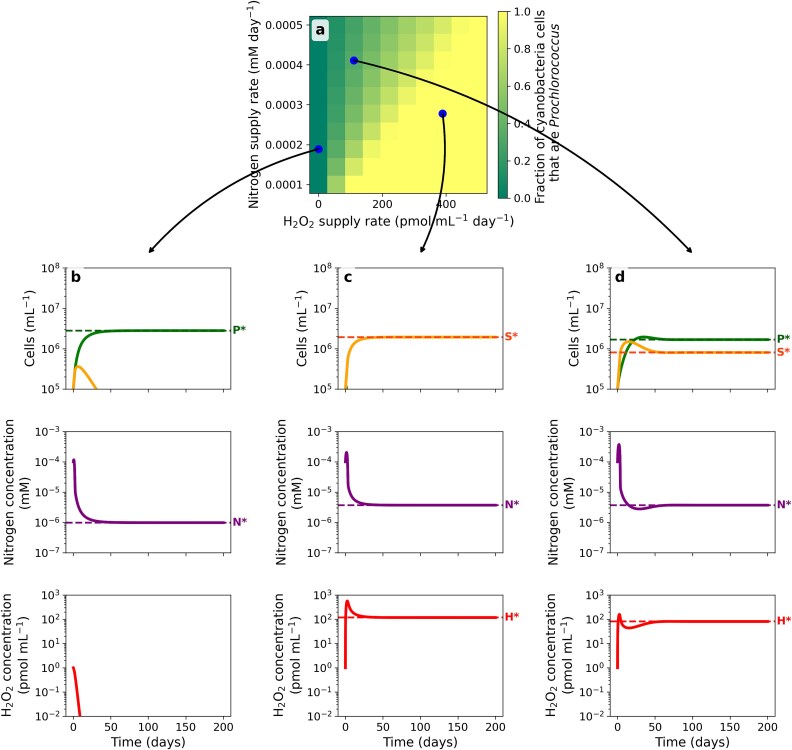
Hydrogen peroxide promotes stable coexistence at high nutrient supply rates. (a) Cyanobacteria taxonomic composition with solutions to Equations 1.1–1.4 (same as Fig. 3b) (b–d) time-dependent solutions for cells (top) nutrients (middle) and H_2_O_2_ concentration (bottom). Horizontal lines in each case are equilibrium solutions given by equations provided in the supplemental information: (a) Equation S2.1.1-S2.1.4, (b) Equation S2.2.1–S2.2.4, and (c) S2.3.1–S2.3.4.

**Figure 5:**
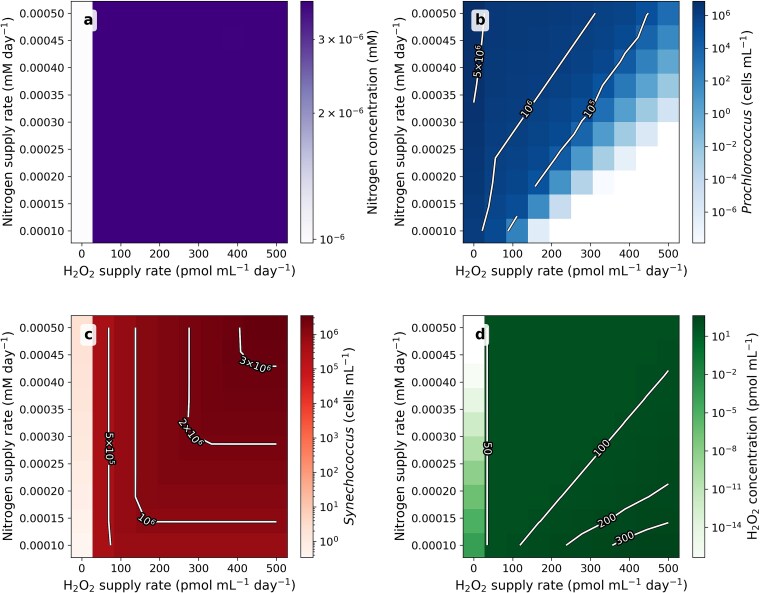
Without heterotrophic bacteria detoxification, unrealistically high *Synechococcus* cell densities (>10^6^ mL^−1^) are required to detoxify hydrogen peroxide to levels tolerable for *Prochlorococcus*. Shown are the solutions to Equations 1.1–1.4 from the simulations reported in Fig. 4 for (a) nutrient, N; (b) *Prochlorococcus*, P; (c) *Synechococcus*, S; and (d) hydrogen peroxide, H. In all cases, white contour lines are benchmarks to indicate environmentally plausible values. Hydrogen peroxide is drawn down to permissible levels when *Synechococcus* far exceed observed concentrations that are maximally ~5 × 10^4^ mL^−1^ [38].

In Fig. 4, we show time-dependent solutions marking the contrasting competitive outcomes depicted in Fig. 3b. In all cases, the system converges upon equilibrium concentrations marked by the horizontal lines and provided in the Supplemental Information (Section S2). In Fig. 5, we show corresponding values for all state variables in Equations 1.1–1.4. Hydrogen peroxide concentration is drawn down to levels permissible for *Prochlorococcus* to coexist with *Synechococcus* (upper left region, Fig. 5d) when *Synechococcus* reaches high cell densities, sustained by high nutrient input (Fig. 5c). Reductions in hydrogen peroxide concentration associated with increased cell density are a consequence of our assumed mass action sensitivity of hydrogen peroxide detoxification in Equation 1.4, which is consistent with laboratory H_2_O_2_ detoxification by *Synechococcus* and other microbes [37]. Interestingly, the *Synechococcus* cell densities required to sustain *Prochlorococcus* in the system far exceed environmental observations, which typically are not greater than ~5 × 10^4^ mL^−1^ [38].

**Figure 6:**
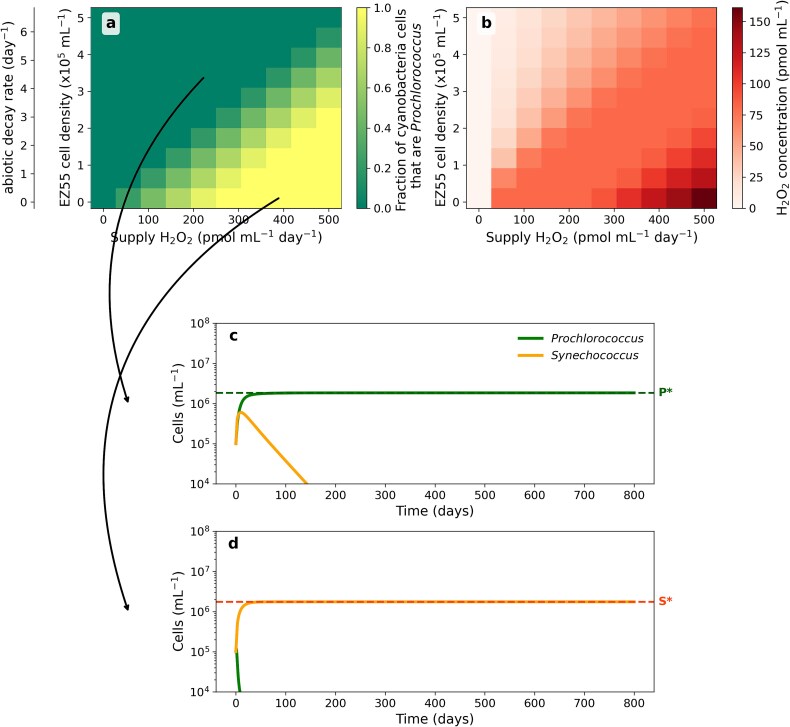
*Prochlorococcus* survival depends critically on the presence of hydrogen peroxide detoxifying heterotrophic bacteria. Shown are cyanobacteria community composition (a) and hydrogen peroxide concentration (b) across a range of heterotrophic bacteria cell densities and hydrogen peroxide supply rates. Note the additional *y*-axis marking the equivalent abiotic decay rate associated with each EZ55 cell density. (c, d) Time-dependent solutions corresponding to the heterotrophic bacteria cell densities and H_2_O_2_ supply rates marked in (a). Modest hydrogen peroxide supply rates are sufficient to eliminate *Prochlorococcus* from the system.

We next conducted simulations to explore the sensitivity of the model to key parameter values: ${k}_{dam}$, ${\phi}_s$, ${\phi}_b$, and ${\delta}_p$. Results of these analyses are reported in the Supplemental Information (Section S3, Figure S4). In Fig. 6, we focus on the sensitivity of the system to biological H_2_O_2_ detoxification and report cyanobacteria composition and time-dependent dynamics for a range of bacteria cell densities. In doing so, we explore the role of an additional community member—a heterotrophic bacterium—maintaining environmentally relevant concentrations of cyanobacteria. Note that for the simulations in Fig. 6, the nutrient supply rate was held at 2.5 × 10^−4^ mM day^−1^—a value within the range assumed in all prior analyses (Table 1, Figs 3 and 4)—and was selected as the supply rate that gave rise to the most ecologically realistic *Prochlorococcus* to *Synechococcus* cell densities. When bacteria cells with detoxification rates equivalent to *Alteromonas macleodii* EZ55 are varied in the range 1 × 10^4^–5 × 10^5^ cells mL^−1^ (Table 1), we find extreme sensitivity of competitive outcomes to hydrogen peroxide supply with hydrogen peroxide supply rates on the order 0–400 pmol mL^−1^ day^−1^ causing a switch from *Prochlorococcus* to *Synechococcus* dominance. These hydrogen peroxide supply rates are well within ranges reported [39]. These findings implicate hydrogen peroxide supply as a determinant of competition among *Prochlorococcus* and *Synechococcus* for a shared limiting resource, in this case ammonium.

Detoxification by heterotrophic bacteria is an undeniable cause of H_2_O_2_ loss [28, 40, 41]. Nevertheless, photochemical H_2_O_2_ decomposition [42] and reactions with copper, iron [43], and other oxidants [44] are important abiotic pathways, all with considerable variability. In this work, we did not attempt to differentiate between various biotic and abiotic degradation pathways. Instead, in Fig. 6a, we mark the equivalent effective abiotic decay rate associated with each bacterial cell density, where equivalence is found by multiplying the bacteria cell density by the mass action decay rate ${\phi}_b$ in Table 1.

## Discussion

Mathematical models have long predicted that exogenous toxins may modify microbial competition outcomes [13–15]. Here, we have introduced ROS to these existing frameworks, and constrained them with data from laboratory experiments and field observations, to ask whether hydrogen peroxide may toggle competition among *Prochlorococcus* and *Synechococcus* for a shared resource. At the core of our analysis is the observation that *Prochlorococcus*’ minimal resource requirement—its “R*”—increases with hydrogen peroxide concentration. Low concentrations of hydrogen peroxide (~80 pmol mL^−1^) may not be high enough to drive ecologically meaningful *Prochlorococcus* mortality, but they are sufficient to toggle its R* just enough for it to lose its competitive advantage against *Synechococcus*. Hydrogen peroxide supply [45] and concentration [46] are both highly dynamic in marine environments, and our simulations indicate this may have important implications for *Prochlorococcus* spatial extent, phenology, and abundance.

Detoxification of H_2_O_2_ is known to be a leaky process, where environmental H_2_O_2_ is detoxified by *Prochlorococcus*’s microbial community members [19]. We modeled leaky community H_2_O_2_ detoxification, first from *Synechococcus* alone, and then from heterotrophic bacteria. Inclusion of leakiness allowed for cell-based drawdown of ambient H_2_O_2_ concentrations in the model, granting *Prochlorococcus* the ability to survive and continue to compete with its community members for nutrients. *Prochlorococcus* rescue was first attempted with *Synechococcus* “help” alone, but the detoxification efficiency of *Synechococcus* was not high enough to allow coexistence of *Prochlorococcus* and *Synechococcus* at realistic abundances (<10^6^ mL^−1^). An additional heterotrophic bacterial helper, an *A. macleodii* analogue, is associated with a much higher detoxification rate than *Synechococcus* [18]. Representation of *A. macleodii*’s detoxifying ability permitted *Prochlorococcus* and *Synechococcus* coexistence in the model, implicating heterotrophic bacteria as ecologically relevant detoxifiers of hydrogen peroxide in marine systems.

By directly influencing *Prochlorococcus* mortality, ROS dynamics may influence marine carbon dynamics in several ways. For example, if H_2_O_2_ drives succession from *Prochlorococcus* to other autotrophs with different growth rates, we would expect to see changes in primary production, and modifications to carbon and nutrient removal. Modifications to carbon removal may be most pronounced if heterotrophic bacteria take advantage of *Prochlorococcus’* diminished ability to compete for resources, as in this instance photosynthesis would be halted, with the potential to shift the balance between autotrophy and heterotrophy. A related question concerns the fate of *Prochlorococcus* cells that are subjected to hydrogen peroxide damage. Dead cells may enter the dissolved or particulate pools, with the potential to be exported from the surface layer or fed upon by heterotrophic bacteria. Future studies may explore the biogeochemical implications of H_2_O_2_-mediated *Prochlorococcus* cell death, but a priority should be to better understand which ocean regimes are associated with most pronounced H_2_O_2_ damage.

The resource competition model we have presented is directly amenable for incorporation within existing ecosystem modeling frameworks, opening the door for future exploration of the impact of hydrogen peroxide on ecosystem properties and ocean biogeochemistry. Nevertheless, numerous extensions to the existing framework are necessary before models can make meaningful predictions about the impacts of ROS on microbial community composition. For example, the heterotrophic detoxifier considered here—*Alteromonas—*is a diverse genus with surface and deep species that differ in their organic C or N usage [47] and pH optima, mirroring the differentiation of *Prochlorococcus* and *Synechococcus* genera through nitrogen, light, and temperature [48–50]. Our simplified model framework did not consider any of this biological diversity. It omitted the potential for heterotrophic bacteria to compete with cyanobacteria for nutrients, and conversely, the potential for *Prochlorococcus* to persist on alternative nitrogen sources, such as amino acids or urea [7, 51]. Expanding the model presented here to account for more diverse community members will require enhanced quantitative understanding of heterotrophic and cyanobacteria resource affinities for a range of inorganic and organic substrates, along with quantitative constraints on susceptibility to grazing [52] and viral infection [53]. Nevertheless, our study will inform future analyses taking a much broader view of the impact of hydrogen peroxide on microbial community composition and function both in the contemporary and ancient ocean [54].

As well as expanding the ecological resolution of the model presented here, future studies will need to more fully resolve processes governing H_2_O_2_ sources and sinks in the marine environment. Our model assumed that hydrogen peroxide is produced photochemically, omitting cell-mediated production [22–24], which has been shown to influence *Prochlorococcus* population dynamics [55]. Our modeling assumed biological activity is a primary driver of H_2_O_2_ degradation. However, non-biological decomposition represents an important and highly variable contributor to H_2_O_2_ loss [42–44]. While beyond the scope of this work, we note that existing remote sensing models of H_2_O_2_ production are ready for implementation in ecosystem modeling contexts [39]. Large-scale spatio-temporal quantification of decay dynamics is more challenging, but some localized datasets could be leveraged for this purpose [25, 44, 45].

Our analysis assumed that the primary impact of hydrogen peroxide on cell physiology is through its damaging effect that arises after diffusion across the cell membrane. This susceptibility varies with environmental conditions like temperature [56] and potentially also light and nutrient availability. However, hydrogen peroxide is also involved in a range of physiological processes [57, 58], for example, influencing cell–cell signaling [59]. Omar et al. (2022) [60] explored the hypothesis that cell signaling in the marine environment may arise when intracellular H_2_O_2_ detoxification causes an extracellular concentration gradient that other microbes sense. However, Omar et al. (2022) [60] concluded that in the marine environment, cells are generally too far apart for the concentration gradient to be detected. In our formulation, intracellular hydrogen peroxide concentration was assumed to impact the average exogenous hydrogen peroxide concentration in a spatially uniform way, thereby providing a benefit to the entire neighboring community.

We assessed *Prochlorococcus* and *Synechococcus* competitive interactions across ranges of nutrient and H_2_O_2_ inputs, reminiscent of ranges experienced by these microbes in surface marine environments [39]. Even at H_2_O_2_ concentrations far below the lethal limit for *Prochlorococcus*, H_2_O_2_ detoxification has an impact on competition between *Prochlorococcus* and *Synechococcus*. At very high cell densities, H_2_O_2_ detoxification by *Synechococcus* and heterotrophic bacteria can lead to coexistence between *Prochlorococcus* and *Synechococcus*. At ecologically relevant cell densities, the impact of H_2_O_2_ detoxification by heterotrophic bacteria determines whether *Prochlorococcus* outcompetes *Synechococcus*. Our modeling assumed a set of fixed environmental conditions with no explicit spatial or temporal variability. It is therefore most applicable to regions of the ocean within limited seasonal variability at low latitudes, where resource competition theory has been shown to provide strong predictions of which phytoplankton dominate [11]. We anticipate future studies conducting more realistic environmental simulations to explore the extent of hydrogen peroxide as a driver of microbial community composition and function.

## Supplementary Material

Supplemental_information_ycag188

## Data Availability

The manuscript contains no new data.
