# Supplementary material for: Resource competition modeling suggests hydrogen peroxide determines competitive outcomes among oligotrophic cyanobacteria
Source: ISME Commun. 2026 Jul 1;6(1):ycag188. doi: 10.1093/ismeco/ycag188 (PMC13431279; doi:10.1093/ismeco/ycag188)
Supplement: Supplemental_information_ycag188 [file supplemental_information_ycag188.docx]

# **Supplemental information for:** Resource competition modeling suggests hydrogen peroxide determines competitive outcomes among oligotrophic cyanobacteria

**Authors:** Donna Katie McCullough, David Talmy

In Section S.1 we report methods for quantification of rates of H_2_O_2_ mediated cell-death for a range of *Prochlorococcus* ecotypes and growth conditions. In Section S.2 we provide equilibrium solutions to the ordinary differential equation model reported in the main text. Section S.3 reports results of a sensitivity analysis exploring changes in cyanobacteria community composition in response to different assumptions about model parameters.

**S.1 Quantification of H_2_O_2_ mediated rates of *Prochlorococcus* cell-death**

Here we outline methodology for quantification of $k_{dam}$, the rate of H_2_O_2_ mediated *Prochlorococcus* cell-death. We expand upon the values of $k_{dam}$ reported by McCullough et al. (2026) by quantifying $k_{dam}$ for different *Prochlorococcus* ecotypes grown in a range of temperature and H_2_O_2_ conditions.

We begin with a model of *Prochlorococcus* growing at their maximal rate ($\mu_{m,p}$) and subjected to cell-death due to hydrogen peroxide following mass-action kinetics with damage rate $k_{dam}$:

| $\frac{dP}{dt} = \underset{growth}{\underbrace{\mu_{m,p}P}} - \underset{\begin{aligned} H2O2 mediated \\ mortality \\ \end{aligned}}{\underbrace{k_{dam}PH}}$ | (S1.1) |
| --- | --- |

McCullough et al. (2025) found that when *Prochlorococcus* is grown in media with ecologically relevant hydrogen peroxide concentrations (~30-90 pmol mL^-1^) the concentration of hydrogen peroxide remains relatively constant, as *Prochlorococcus’s* limited ability to detoxify H_2_O_2_ balances H_2_O_2_ produced in culture. We therefore assumed that concentration is fixed at $H_{m}$. With this simplification, and assuming an initial *Prochlorococcus* cell density $P_{0}$, Equation S1.1 can be solved analytically:

| $P\left( t \right)=$ $P_{0}e^{{(\mu}_{m,p}-k_{dam}H_{m})t}$ | (S1.2) |
| --- | --- |

After log-transformation, we find:

| $ln(P\left( t \right))=$ ${ln(P}_{0})+{(\mu}_{m,p}-k_{dam}H_{m})t$ | (S1.3) |
| --- | --- |

Equation S1.3 is a straight-line relating population density with time, t. Its form provides a suitable representation of variation in cell density for a range of fixed hydrogen peroxide concentrations (McCullough et al., 2026). The intercept ${ln(P}_{0})$ can be retrieved by fitting equation S1.3 to log-transformed measurements of *Prochlorococcus* dynamics through time, but the slope has three unknown values $\mu_{m,p},k_{dam}$, and $H_{m}$. The hydrogen peroxide concentration ($H_{m})$ was measured by Ma et al. (2018) for high light *Prochlorococcus* ecotypes MED4 and MIT9312, leaving two unknowns in Equation S1.3: $\mu_{m,p}$ and $k_{dam}$. In experiments by Ma et al., (2018), the fixed hydrogen peroxide concentration was measured within the range 0 to 400 pmol mL^-1^. In the special case with no H_2_O_2_ present, Equation S1.3 reduces to:

| $ln(P\left( t \right))=$ ${ln(P}_{0})+\mu_{m,p}t$ | (S1.4) |
| --- | --- |

and the maximal growth rate $\mu_{m,p}$ can be quantified directly through line fitting. In this way, quantification of $\mu_{m,p}$and $H_{m}$ through a combination of modeling (for the maximal growth rate, $\mu_{m,p}$) and measurement (for the hydrogen peroxide concentration, $H_{m}$) leaves one unknown in Equation S1.3: $k_{dam}$, which can be quantified by fitting Equation S1.3 to measurements of log-transformed cell density through time.

In Figure S1.1 and S1.2, we report log-transformed cell densities of *Prochlorococcus* ecotypes MED4 and MIT9312, with lines fit through ordinary least squares regression, for a range of temperatures and hydrogen peroxide concentrations. In Figure S1.3, we summarize the values of $k_{dam}$ that are consistent with the experimental data and the model in Equation S1.3. The $k_{dam}$ values for MED4 grown at 28°C and MIT9312 grown at 22°C at the lowest H_2_O_2_ concentrations (61 and 90 pmol mL^-1^ for MED4 and MIT9312, respectively) are both 0.002 mL pmol^-1^ day^-1^. This value represents a modest reduction in *Prochlorococcus* net growth rate in response to an environmentally relevant concentration of hydrogen peroxide, at the edge of the temperature range these high light ecotypes have evolved to tolerate, and is the value assumed in all simulations reported in the main text.

| 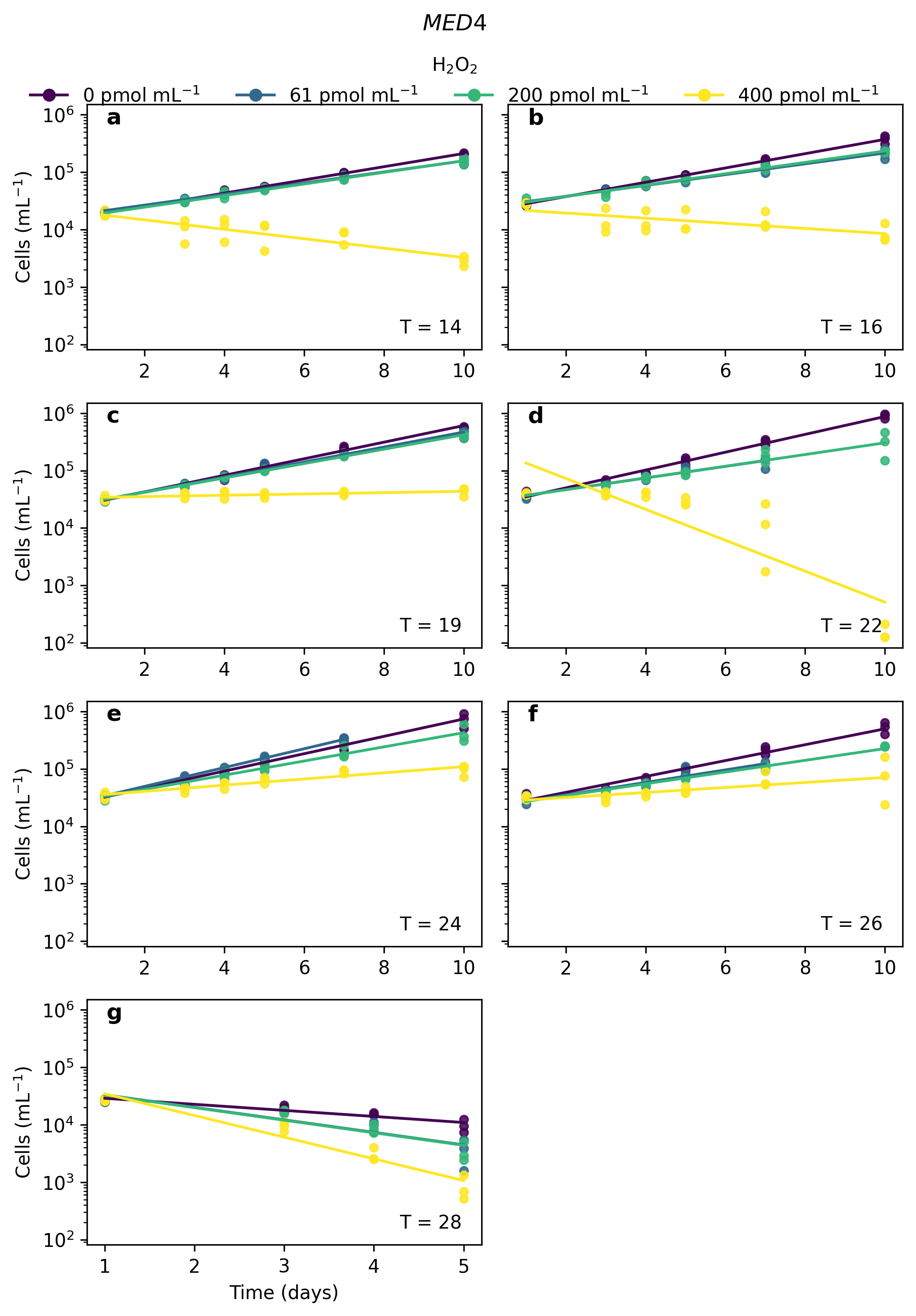 |
| --- |
| **Figure S1:** *Prochlorococcus* MED4 population dynamics in batch culture experiments (symbols) from Ma et al 2018. Lines were fitted to experimental data with ordinary least squares regression. |

| 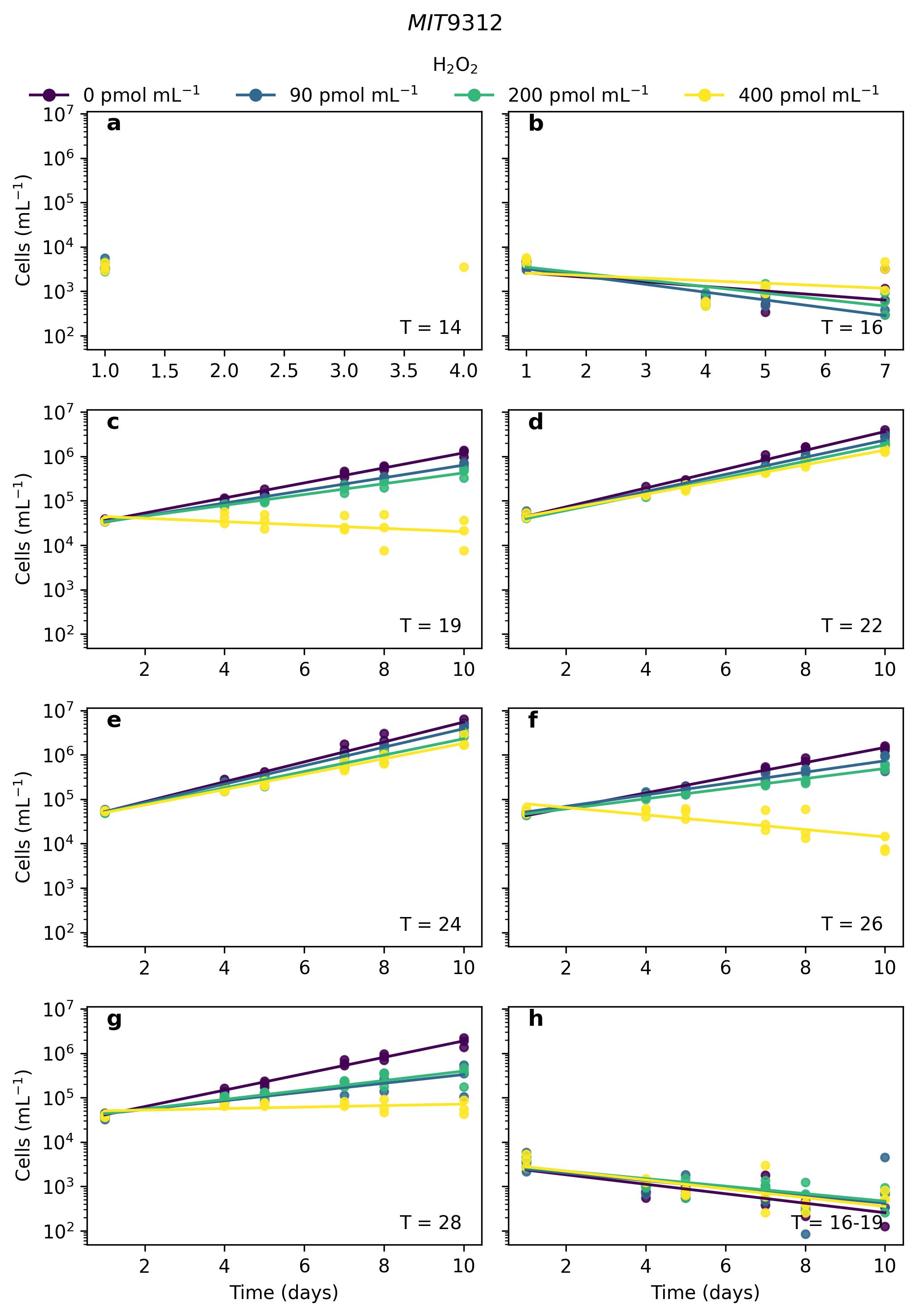 |
| --- |
| **Figure S2:** *Prochlorococcus* MIT9312 population dynamics in batch culture experiments (symbols) from Ma et al 2018. Lines were fitted to experimental data with ordinary least squares regression. |

| 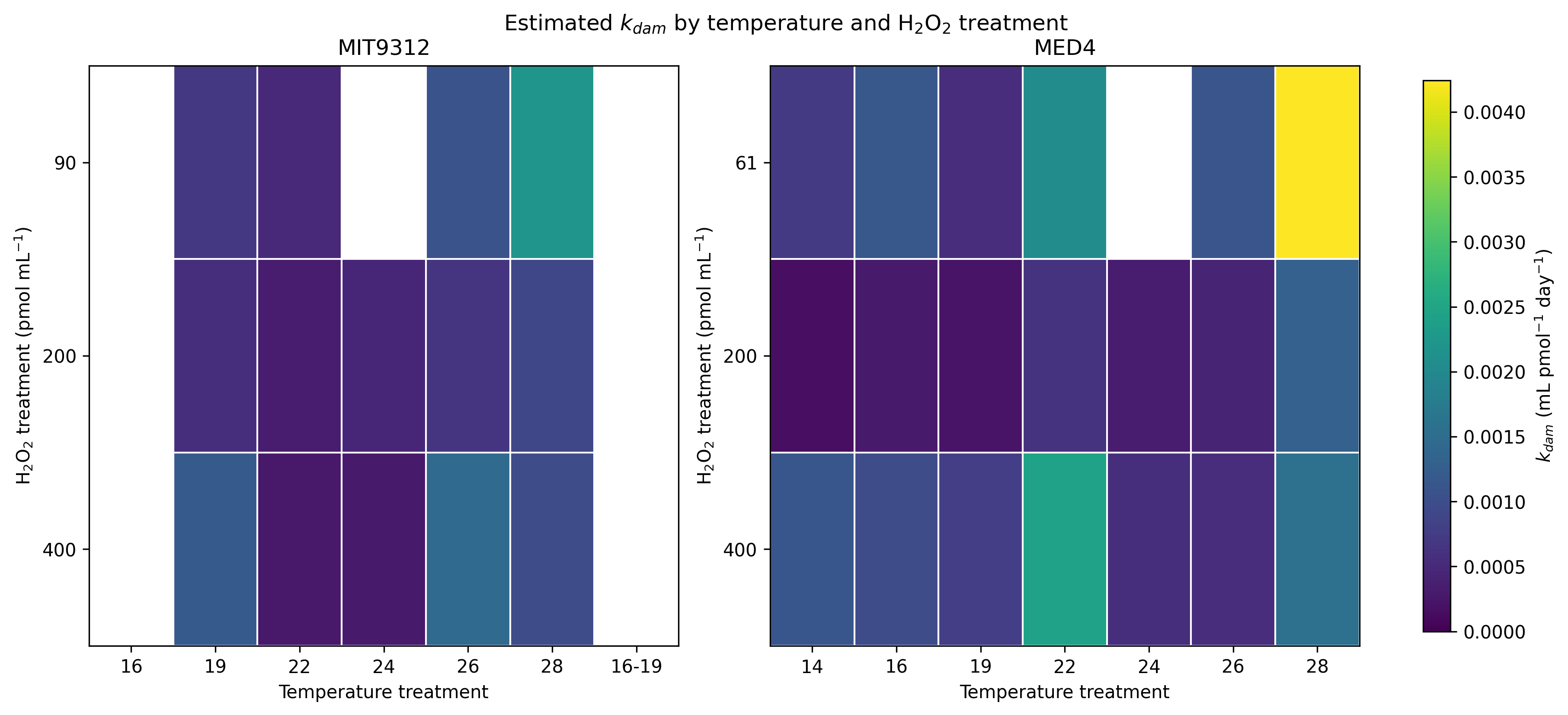 |
| --- |
| Figure S3: Heatmap of H_2_O_2_ mediated rates of *Prochlorococcus* cell death (i.e. $k_{dam}$) across a range of temperature and H_2_O_2_ conditions for MIT9312 (left) and MED4 (right). Values were inferred by combining the slopes of the lines in Figures S1 and S2 with Equation S1.3. |

**S.2 Equilibrium solutions**

Here we provide the steady state solutions to the system of Equations 1.1-1.4 (see main text), found by setting each Equation to zero and solving for each state variable. We provide the three ecologically relevant set of states, corresponding to *Prochlorococcus* and *Synechococcus* coexistence (S2.1.1-S2.1.4) and cases where either cyanobacterium outcompetes the other (S2.2.1-S2.2.4 and S2.3.1-S2.3.4). Each set of equilibria mark the horizontal lines marked in Figures 4 and 6. We do not provide the trivial cases where neither *Prochlorococcus* and *Synechococcus* survive.

*Coexistence*

| $N^{*} =\frac{K_{s,s}\delta_{s}}{\mu_{m,s}-\delta_{s}}$ | (S2.1.1) |
| --- | --- |
| $P^{*} = \frac{1}{Q_{n,p}}\frac{N^{*}+K_{s,p}}{\mu_{m,p}N^{*}}\left( \lambda_{n}-Q_{n,s}\frac{\mu_{m,s}N^{*}}{N^{*}+ K_{s,s}}S^{*}-\delta_{n}N^{*} \right)$ | (S2.1.2) |
| $S^{*} = \frac{1}{\phi_{s}}\left[ \frac{\lambda_{h}}{H^{*}} -(\phi_{b}B^{*}+\delta_{h}) \right]$ | (S2.1.3) |
| $H^{*} = \frac{1}{k_{dam}}\left[ \frac{\mu_{m,p}N^{*}}{N^{*}+ K_{s,p}} -\delta_{s} \right]$ | (S2.1.4) |

Prochlorococcus *outcompetes* Synechococcus

| $N^{*} =\frac{K_{s,p}(\delta_{p}+k_{dam}H^{*})}{\mu_{m,p}-(\delta_{p}+k_{dam}H^{*})}$ | (S2.2.1) |
| --- | --- |
| $P^{*} =\frac{1}{Q_{n,p}}\frac{N^{*}+K_{s,p}}{\mu_{m,p}N^{*}}\left( \lambda_{n}-\delta_{n}N^{*} \right)$ | (S2.2.2) |
| $S^{*} = 0$ | (S2.2.3) |
| $H^{*} = \frac{\lambda_{h}}{\phi_{b}B^{*}+\delta_{h}}$ | (S2.2.4) |

Synechococcus *outcompete* Prochlorococcus

| $N^{*} =\frac{K_{s,s}\delta_{s}}{\mu_{m,s}-\delta_{s}}$ | (S2.3.1) |
| --- | --- |
| $P^{*} = 0$ | (S2.3.2) |
| $S^{*} =\frac{1}{Q_{n,s}}\frac{N^{*}+K_{s,s}}{\mu_{m,s}N^{*}}\left( \lambda_{n}-\delta_{n}N^{*} \right)$ | (S2.3.3) |
| $H^{*} = \frac{\lambda_{h}}{\phi_{s}S^{*}+\phi_{b}B^{*}+\delta_{h}}$ | (S2.3.4) |

**S.3 Sensitivity analysis**

To explore the robustness of these findings to different assumptions about model parameter values, we repeated the simulations reported in Figures 4 and 5 (main text) independently increasing and decreasing four key parameters: i) the rate of H_2_O_2_ induced cell death ($k_{dam}$), ii-iii) *Synechococcus* and heterotrophic bacteria cell specific detoxification rates ($\phi_{s}$ and $\phi_{b}$, respectively), and iv) the rate of background mortality for *Synechococcus* and *Prochlorococcus* ($\delta_{p}$). Results of these analyses are reported in Figure S4. Across all simulations, the same basic relationship persists, with coexistence maintained when hydrogen peroxide is produced, and nutrient supply rate is sufficiently high to sustain a sizeable *Synechococcus* population. When hydrogen peroxide is present, increases in damage rates and rates of cell death increase the level of nutrient supply required for *Synechococcus* and *Prochlorococcus* to coexist, and increases in rates of detoxification have the opposite effect, decreasing the minimal nutrient supply rate required for coexistence.

| 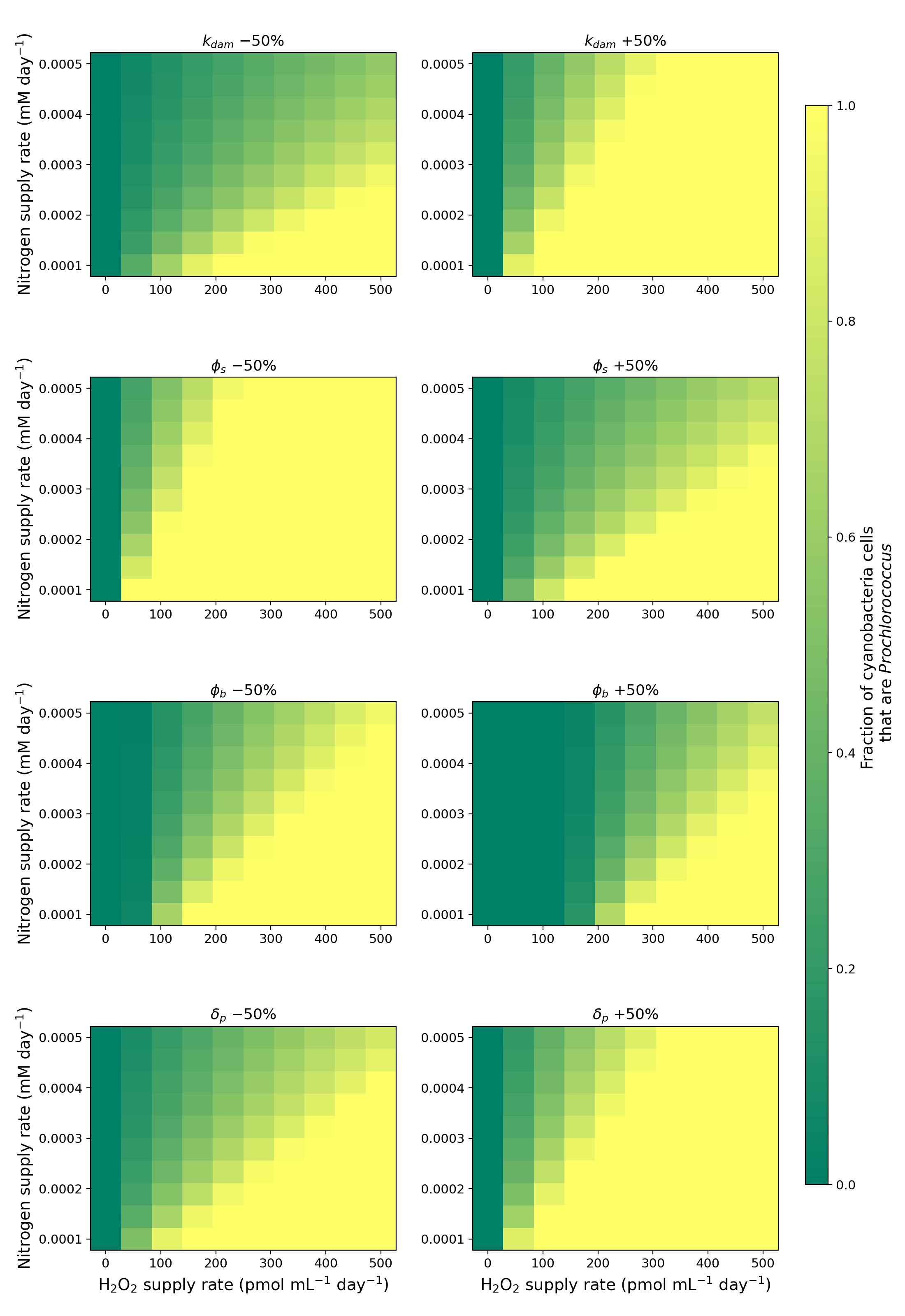 |
| --- |
| Figure S4: Sensitivity of modeled cyanobacteria community composition in response to different assumptions about the rate of H_2_O_2_ mediated cell death ($k_{dam}$, top row), *Synechococcus* and heterotrophic bacteria cell specific detoxification rates ($\phi_{s}$ and $\varphi_{b}$, 2^nd^ and 3^rd^ rows, respectively), and rates of background mortality for *Synechococcus* and *Prochlorococcus* ($\delta_{p}$, bottom row). The left and right columns are 50% decrease and increase relative to the parameters noted in Table 1 of the main text. |
